# Non-intubated uniportal subxiphoid thoracoscopic extended thymectomy for thymoma associated with myasthenia gravis

**DOI:** 10.1186/s12957-021-02430-z

**Published:** 2021-12-09

**Authors:** Zhengcheng Liu, Louqian Zhang, Weifeng Tang, Rusong Yang

**Affiliations:** Department of Cardiovascular and Thoracic Surgery, Nanjing Drum Tower Hospital, The Affiliated Hospital of Nanjing University Medical School, Nanjing, 210029 China

**Keywords:** Non-intubated, Uniport, Subxiphoid, Thymectomy, Myasthenia gravis

## Abstract

**Background:**

To describe a technique of non-intubated uniportal subxiphoid thoracoscopic extended thymectomy.

**Methods:**

Data were collected retrospectively. A single 3-cm transverse incision was made below the xiphoid process. This method for extended thymectomy entails adoption of uniportal subxiphoid VATS combined with using of non-intubated anesthesia for thymoma associated with myasthenia gravis.

**Results:**

Ten consecutive patients underwent this procedure successfully. Mean operative time was 102.5 min. Conversion to intubated ventilation or thoracotomy was not required.

Mean chest tube duration was 3.5 days. Mean postoperative hospital stay was 4.7 days. Histologic examination showed early-stage thymomas. Side effects were rare. Quantitative MG scores decreased during follow-up.

**Conclusions:**

Patients were uneventfully discharged with fast recovery. This technique may merge the potential benefits of a subxiphoid incision and the non-intubated anesthesia protocol.

## Introduction

The traditional method for surgical treatment of thymoma with myasthenia gravis (MG) is extended thymectomy through median sternotomy [1]. Uniportal subxiphoid thoracoscopic approach may be more optimized way to proceed in the operating room with less intercostal neuralgia, faster recovery, and lower cost. In addition, the operative visualization is improved with access to the anterior mediastinum space, including the bilateral phrenic nerves and great vessels [2]. Non-intubated anesthesia might prevent injury of the laryngopharynx and trachea caused by intubation and extubation, it might be beneficial by reducing postoperative myasthenic crisis as well [3].

We describe a novel technique of non-intubated uniportal subxiphoid thoracoscopic extended thymectomy.

### Patients and methods

The application of this new technique was approved by the institutional review board, and all patients provided written informed consent before operation. Between December 2018 and May 2021, 10 cases of thymoma associated with myasthenia gravis were observed and treated.

Patients considered appropriate for this technique met the following criteria: age 18–65, preoperative diagnosis of thymoma with Myasthenia Gravis Foundation of America (MGFA) class I–II, American Society of Anaesthesiologists (ASA) grade of I–II, Masaoka stage I–II with maximal diameter < 5 cm. Patients with a bleeding disorder, overweight(BMI > 28), pulmonary insufficiency, history of chest surgery and potential difficult airway for intubation were not considered suitable.

Patient was kept in lithotomy position. The anesthesia protocol was previously reported [2]. In brief, after intravenous infusion of dexmedetomidine 1 μg/kg by pump injection (completed within 15 min), anesthesia was induced with intravenous dexamethasone 10.00 mg, midazolam 0.10 mg/kg and sufentanil 0.1–0.2μg/kg, with the target-controlled infusion (TCI) of propofol (target plasma concentration of 2–3 μg/ml). To keep oxygen saturation above 95%, pre-lubricated laryngeal mask was inserted for spontaneous ventilation with 100% inspired oxygen (4–5 L/min). Skin infiltration was performed at operation site with 0.375% ropivacaine and 1.00% lidocaine. Maintenance of anesthesia was done with TCI of propofol (target plasma concentration of 1–2 μg/ml), dexmedetomidine (0.5–1 μg/kg/h) and remifentanil (0.1 ~ 0.5 ug/kg/h). We used train-of-four (TOF) monitoring during surgery and ensured full recovery prior to terminating the anesthetics. BIS monitoring was maintained at 40–60 during the operation.

A single 3-cm transverse incision was made below the xiphoid process. The retrosternal space was opened by blind blunt dissection. Plastic wound protector was used. A gradual and natural collapse of the lung occurred after opening pleural cavity.

**Fig. 1:**
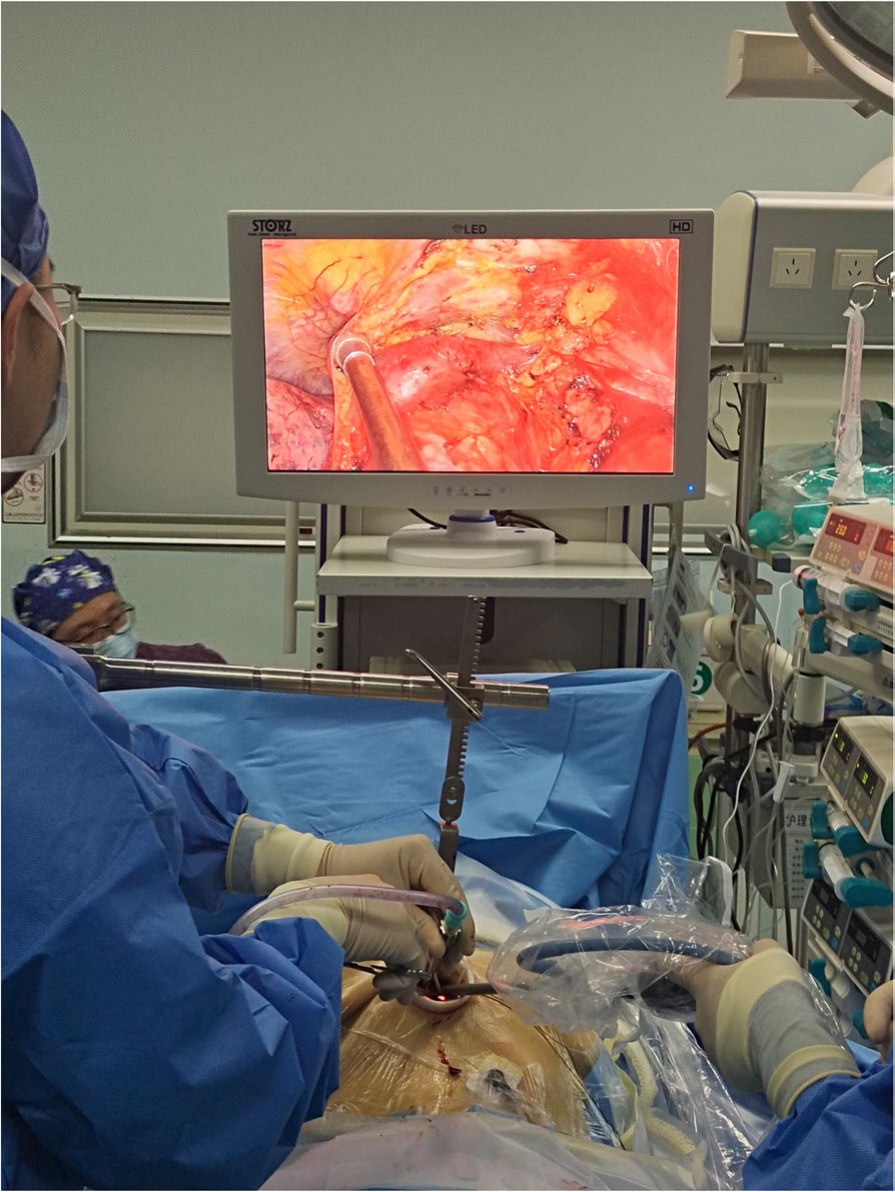
A single transverse incision was made below the lower edge of the xiphoid

During operation, a 10-mm 30° thoracoscope and several thoracoscopic instruments could be simultaneously fitted into the uni-port. A sternum retractor would be placed at the incision site to raise the sternum about 2 cm and facilitate surgical maneuvers (Fig. 1).

**Fig. 2:**
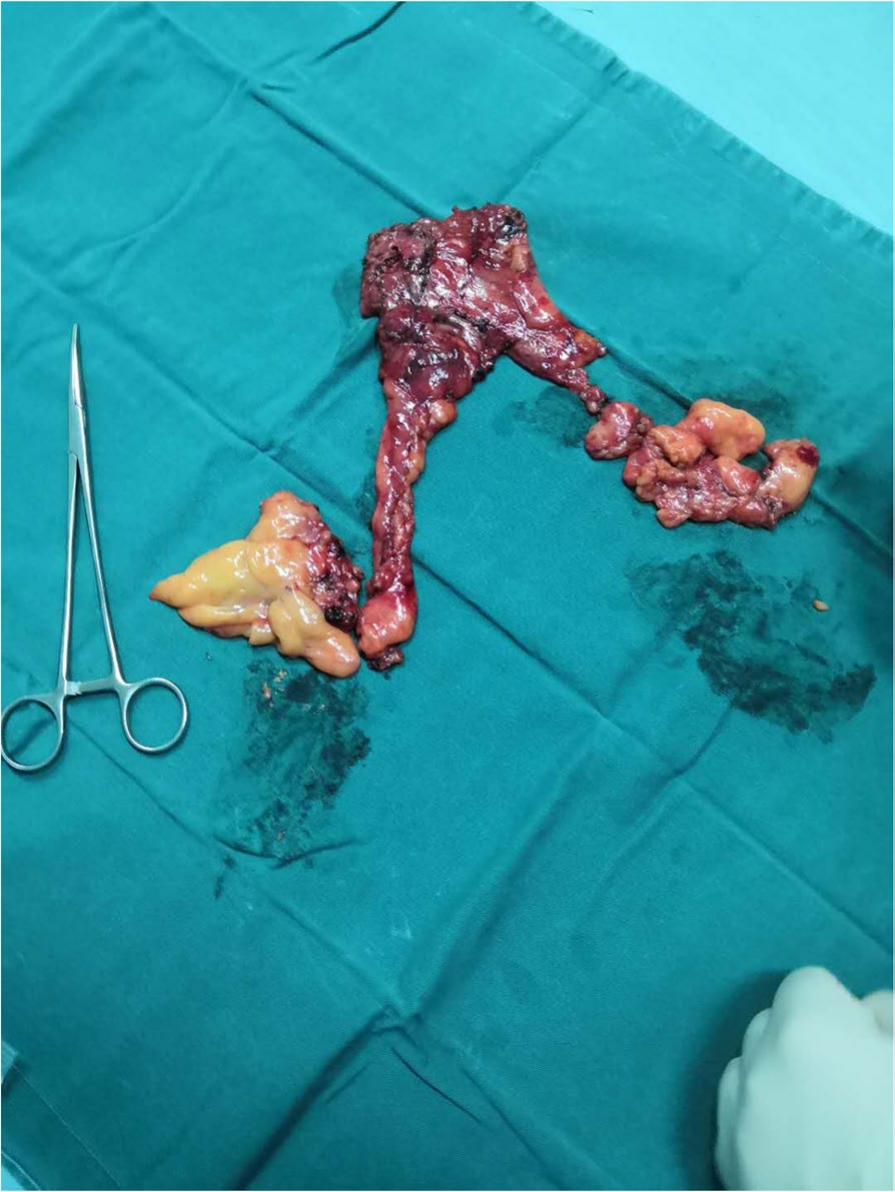
Radical removal of the lesion and the entire thymus gland with fat tissue was accomplished

The following principles should be observed during the case. Both sides of mediastinal pleura should be opened to the level of the internal thoracic vessels. When lower edge of the thymus and the connecting pericardial fat was visualized, a thymectomy was started at the level of the diaphragm and proceeded gradually in an upward direction. Phrenic nerve should be identified and carefully protected. Thymic veins were ligated using ultrasonic scalpe, electric cautery or an appropriately sized clip, then the upper horns of the thymus was isolated. Dissection was continued above the innominate vein to fossa suprasternalis, radical removal of the tumor and the entire thymus gland, including perithymic fatty tissue between both phrenic nerves, from cardiophrenic angles inferiorly to the lower cervical region superiorly was accomplished to achieve an extended thymectomy (Fig. 2). Lymph nodes in anterior mediatinum were sampled. Specimen was placed into a bag and removed through the incision protector.

At the end of the operation, a single-lumen central venous catheter (7 Fr, 20 cm) was inserted in each side of thoracic cavity at the end of the operation. All patients were transferred to the post anesthesia care unit for recovery.

## Results

Ten consecutive patients underwent this procedure successfully. The clinical features were listed in Table 1. MGFA class was I in 5 cases, IIA in 4 cases, stage IIB in 1 case. All patients took pyridostigmine preoperatively, 1 patients took steroids. Mean operative time was 102.5 min. The detectable lowest mean SpO_2_ and EtCO_2_ during operation was acceptable. Conversion to intubated ventilation or thoracotomy was not required.

**Table 1:** Peri-operative data of patients

	Patient 1	Patient 2	Patient 3	Patient 4	Patient 5	Patient 6	Patient 7	Patient 8	Patient 9	Patient 10
Sex	Male	Male	Female	Male	Female	Female	Male	Female	Female	Male
Age	41	55	39	51	53	36	58	43	36	45
FEV1(L)	2.94	3.11	2.52	2.77	2.98	2.85	3.38	2.46	3.02	3.46
Smoking status	Cessation	No	No	Cessation	No	No	Cessation	No	No	No
Body mass index (kg/m^2^)	23.7	22.4	25.1	24.2	23.9	22.6	23.5	22.1	24.7	23.5
ASA score	1	1	1	1	1	1	1	1	1	1
Thoracic adhesion	No	No	No	Mild	No	No	No	No	No	No
Operation time (min)	95	105	115	125	90	95	100	85	110	105
Chest tube drainage (days)	3	3	5	4	3	4	3	4	3	3
Postoperative hospital stay (days)	4	5	7	5	4	5	4	5	4	4
Pain in postoperative day 1 (VAS)	2	3	1	2	2	2	2	1	1	2
Pain in postoperative day 3 (VAS)	2	3	1	2	1	1	2	1	1	1
Pain in postoperative day 7 (VAS)	1	1	1	1	1	1	1	1	1	1
Myasthenia gravis classification (before operation)	IIB	IIA	I	I	IIA	IIA	I	I	IIA	I
Preoperative QMG score	6	6	2	3	5	5	2	3	5	3
Preoperative duration of symptoms (months)	9	3	2	1	2	6	2	3	2	12
Pathology type	AB	B1	A	B2	AB	B2	B1+B2	B1	B1	AB
Masaoka stage	I	IIA	I	IIA	I	IIA	IIA	I	IIA	I
Maximal diameter (mm)	29	22	19	45	33	23	35	28	30	26
Lymph node invasion	No	No	No	No	No	No	No	No	No	No
Follow-up (months)	21	19	17	16	13	12	10	9	9	8
Postoperative QMG score (6 months)	1	2	2	1	2	2	2	1	3	1
Myasthenic crisis	No	No	No	No	No	No	No	No	No	No
Myasthenia gravis status (after operation)	Improved	Improved	Improved	Improved	Improved	Improved	Improved	Improved	Improved	Improved
Thymoma recurrence	No	No	No	No	No	No	No	No	No	No

The postoperative course was uneventful. Mean chest tube duration was 3.5 days. Mean postoperative hospital stay was 4.7 days. Histologic examination showed early-stage thymomas with R0 resection. The postoperative pain VAS score was low (1–3). Side effects were rare, pleural effusion was found in 3 cases, thoracentesis was not needed. Quantitative MG scores decreased with improved myasthenia gravis status during follow-up. The median (range) follow-up period was 16 (4–33) months, tumor recurrence or myasthenic crisis was not occurred. Outcomes of the patients are detailed in Table 1.

## Discussion

With a combination of uniportal subxiphoid access and non-intubated anesthesia, this method resulted in minimal invasiveness and good perioperative outcomes.

Postoperative myasthenia crisis after thymectomy was reported from 6 to 34% [3]. In patients with MG, response to muscle relaxants was usually unpredictable with decreased neuromuscular transmission, the susceptibility to postoperative respiratory failure was also increased. Avoiding using muscle relaxants and intubation may lead to relief of symptoms, reduction of complications and fast recovery. In this series, quantitative MG scores were decreased with improved myasthenia gravis status during follow-up.

Visualization of the entire anterior mediastinum space up to the lower cervical area was highly satisfactory during operation [4, 5]. Extended thymectomy with dissection of fat tissue could be achieved using this approach.

The oxygen saturation might be low with high peak EtCO_2_ level. We use a laryngeal mask as a safety precaution for ventilatory management to maintain satisfactory oxygenation during spontaneous respiration, even after opening of both mediastinal pleura [2, 6].

VAS scores were low, a single-lumen central venous catheter was placed in each side of thoracic cavity instead of one chest drain inserted to anterior mediastinal space. It was sufficient for drainage, and it caused much less postoperative pain with better wound healing. Side effects were rare in this series, pain was mild, and patients might recover better using this technique.

The duration of postoperative hospital stay was relatively long. Patients in China usually do not have to pay high costs for prolonged hospitalization, and they usually prefer to stay in the hospital for an additional 1 or 2 days after an operation despite having met the criteria for discharge.

During our preoperative counseling, we discussed with patients and showed the benefits and risks of non-intubated anesthesia and uniportal subxiphoid thoracoscopic surgery to patients, conversion to traditional intubation anesthesia or thoracotomy was prepared and could be applied as soon as it was needed (uncontrolled bleeding, tumor invasion to great vessel, unstable ventilation et al.), in order to relieve anxiety of patients. There were also some risks of this procedure, laryngeal mask might also encounter associated complications such as gastric insufflation, reflux or mask displacement. Bleeding control was hard in uniportal subxiphoid surgery, additional incision or thoracotomy was needed in these cases.

There were several limitations to this present study. For retrospective nature of the study, the randomization was absent, and selection bias cannot be eliminated. Prospective research was needed to further confirm the conclusion. Small sample size and short follow-up time were also the main limitations. Long-term and subjective patient outcomes should be established in future studies.

## Conclusions

Extended thymectomy through a single subxiphoid incision without intubation was feasible for thymoma with MG. It might be beneficial by reducing postoperative myasthenic crisis with mild pain and fast recovery, and prevent injury of the laryngopharynx and trachea caused by intubation and extubation.

## Data Availability

The datasets used and/or analyzed during the current study are available from the corresponding author on reasonable request.

## Abbreviations

VATS: Video-assisted thoracoscopic surgery
MGFA: Myasthenia Gravis Foundation of America
ASA: American Society of Anaesthesiologists
TOF: Train-of-four monitoring
